# Immunomodulator-Based Enhancement of Anti Smallpox Immune Responses

**DOI:** 10.1371/journal.pone.0123113

**Published:** 2015-04-13

**Authors:** Osmarie Martínez, Eric Miranda, Maite Ramírez, Saritza Santos, Carlos Rivera, Luis Vázquez, Tomás Sánchez, Raymond L. Tremblay, Eddy Ríos-Olivares, Miguel Otero

**Affiliations:** 1 Department of Microbiology, University of Puerto Rico, Medical Sciences Campus, San Juan, Puerto Rico; 2 Department Biology, University of Puerto Rico, Rio Piedras Campus, San Juan, Puerto Rico; 3 Department of Microbiology Universidad Central del Caribe School of Medicine, Bayamón, Puerto Rico; 4 Department of Biology, University of Puerto Rico, Humacao, Puerto Rico; 5 Department of Biology, University of Puerto Rico, Rio Piedras Campus, San Juan, Puerto Rico; 6 Center for Applied Tropical Ecology and Conservation, University of Puerto Rico, Rio Piedras campus, San Juan, Puerto Rico; Imperial College London, UNITED KINGDOM

## Abstract

**Background:**

The current live vaccinia virus vaccine used in the prevention of smallpox is contraindicated for millions of immune-compromised individuals. Although vaccination with the current smallpox vaccine produces protective immunity, it might result in mild to serious health complications for some vaccinees. Thus, there is a critical need for the production of a safe virus-free vaccine against smallpox that is available to everyone. For that reason, we investigated the impact of imiquimod and resiquimod (Toll-like receptors agonists), and the codon-usage optimization of the vaccinia virus A27L gene in the enhancement of the immune response, with intent of producing a safe, virus-free DNA vaccine coding for the A27 vaccinia virus protein.

**Methods:**

We analyzed the cellular-immune response by measuring the IFN-γ production of splenocytes by ELISPOT, the humoral-immune responses measuring total IgG and IgG2a/IgG1 ratios by ELISA, and the TH1 and TH2 cytokine profiles by ELISA, in mice immunized with our vaccine formulation.

**Results:**

The proposed vaccine formulation enhanced the A27L vaccine-mediated production of IFN-γ on mouse spleens, and increased the humoral immunity with a TH1-biased response. Also, our vaccine induced a TH1 cytokine milieu, which is important against viral infections.

**Conclusion:**

These results support the efforts to find a new mechanism to enhance an immune response against smallpox, through the implementation of a safe, virus-free DNA vaccination platform.

## Introduction

Smallpox is a disease caused by variola virus, which is a complex, enveloped, double-stranded DNA virus. There are two clinical forms of this virus, the first is variola major, which has the capacity to cause a more complicated illness and higher mortality compared to the other form, the variola minor [1]. Smallpox was eradicated in 1980 through a global vaccination effort administered by the World Health Organization (WHO) [2]. After the eradication, the scientific community agreed to destroy the stockpiles of the virus and currently only two official stores of variola exist [3]. However, access to variola virus could be easier than expected, not only because other viral stocks might be stored elsewhere [4], but also because of the possibility of isolating the virus from corpses buried in the Siberian permafrost, of people who died of smallpox infection [4].

Smallpox is a threat to public health in the event that the virus reappears in the population [5, 6]. The current most efficient tool against this agent is the licensed live vaccinia virus vaccine. However, complications such as active myocarditis, encephalitis [7–10], progressive vaccinia [7], severe skin infections [7, 11], and even death [7, 11] have been observed after administering this vaccine. Moreover, the vaccine is contraindicated for [12] immune-compromised individuals [13], transplant recipients [14, 15], patients under immune-suppressive therapy [14, 15], and pregnant women [16]. Implementation of a massive vaccination campaign with the current vaccine could be devastating.

DNA-based vaccines have shown to produce antigen-specific humoral- and cellular-immune responses in several organisms [17–19]. They are safe as they are non-live, non-spreading and non-replicating [12, 20–22]. As it is the host that is producing the antigenic protein of interest [19, 23], the antigens will have those post-translational modifications produced during a real infection [19, 23]. DNA vaccines have been used in clinical trials [24–26] with no adverse events. They have a long-term shelf life, do not need to be stored at low temperatures, and are easy to produce as they can be generated in bacteria. These properties make DNA immunization a promising methodology for vaccine development against viral infections.

In many cases, immunizing with the DNA alone is not enough to trigger an optimal immune response; for that reason, the use of an adjuvant is necessary. We test imiquimod and resiquimod as the adjuvants in our vaccine design, formulated in a cocktail with a plasmid DNA coding for the A27 protein of the Vaccinia Virus Western Reserve (VVWR) strain. A27 is a 14-kDa envelope protein that is conserved in the poxviruses [27] and known to induce cell- and humoral-mediated immune responses in mice [28, 29].

Imiquimod is an imidazoquinoline amine approved for the topical treatment of external genital warts [30]. It functions as an immune response modifier that in animal models has shown to induce potent antiviral and antitumor activities [31]. Besides other cytokines, it induces the expression of IFN-α, which has an impact in the production of IL-12 and IFN-γ [31]. Its mechanism is based on the activation of immune cells via the TLR-7 MyD88-dependent pathway [32]. Imiquimod has been tested in several clinical trials against diseases like neoplasia [33, 34] and Herpes Simplex Virus 2 infection [35]. Resiquimod is a chemical analog of imiquimod that uses the same mechanism of immune activation of imiquimod. Resiquimod has been used in clinical studies for the treatment of genital herpex [36], viral skin lesion, and skin cancer [37].

As stated before, the cytokine milieu generated during the innate responses has a role tailoring the adaptive responses [38–40]. For this reason, targeted activation of Toll-like receptors is one of our major research interests. In this regard, our previous data shows that resiquimod (an analog of imiquimod) generates a TH1 cytokine milieu, after vaccination with plasmid HIV-gag, inducing a cell-mediated immune response [41].

Based on our previous work, in this study we compared the effect of imiquimod (FDA approved treatment against venereal warts) and resiquimod in combination with DNA at a concentration of 1mg/mL (formulated in a buffer of 0.15 mM citrate and 0.25% bupivacaine). Our vaccines are formulated in 50 nmoles of imiquimod, which is a Toll-like receptor-7 (TLR-7) agonist [39] or 50 nmoles of resiquimod, which is a Toll-like receptor 7 and 8 agonist [42]. We show that the intramuscular (i.m.) administration of our vaccine, formulated in imiquimod or resiquimod, induce a VVWR A27-specific cell-mediated response in a mouse model, which is known to be effective against viral infections.

Codon optimization is an approach proven to enhance the immune responses of DNA vaccines, presumably by increasing antigen expression in the host. After implementing this approach, our data shows that codon-optimization has a positive impact in our DNA vaccine, as we observe an improvement in the antigen-specific immune responses in animals immunized with the vaccine formulated with codon-optimized DNA. We expect to provide additional information for the optimization of antigen engineering and vaccine formulation. A rational vaccine design will offer guidance to successfully modulate the innate immune response against bioterror agents, as well as emerging infectious diseases.

## Materials and Methods

### Ethics statement

Female 4–6 week-old BALB/c mice were purchased from Charles River (Wilmington,MA, USA). Care of the animals was in accordance with the guidelines from The National Institutes of Health (Bethesda, MD, USA). This protocol was approved by the University of Puerto Rico Institutional Care and Use Committee (IACUC) (Approval Number 9250112). Mice were anesthetized by intraperitoneal injection with a mixture of Ketamine and Xylazine, and humanely euthanized via cervical dislocation to permit analysis of immune responses. This method is consistent with the recommendations of the Panel on Euthanasia of the American Veterinary Medical Association. All efforts were implemented to minimize pain and suffering.

### Design of the VVWR DNA vaccine

The A27L gene from Vaccinia Virus Western Reserve (VVWR) used to generate our vaccination construct (pA27L) was synthesized by BlueHeronBio (Bothell, WA, USA), and cloned into the pVax1 (Invitrogen, Grand Island, NY, USA) HindIII and NotI (New England Biolabs, Ipswich, MA, USA) sites. The clone contains an IgE leader sequence for secretion, and a hemagglutinin tag sequence for expression and additional characterization, if necessary.

### Gene optimization

The A27L gene from VVWR was sequenced-optimized using the GeneOptimizer Process by Life Technologies (Grand Island, NY, USA), to generate the pA27LOPT construct used as a vaccine in this study. Modifications included the removal of the sequence repeats, adjustment of the codon usage to mouse, GC content optimization, elimination of the killer motifs, removal of the splice sites and decrease of possible RNA secondary structures. The pA27LOPT construct was also subcloned into the pVax1 vector in the same manner as the A27L gene described above.

### Plasmid expansion and purification

The pA27L and pA27LOPT constructs were transformed individually in *E*.*coli* Top10 (Invitrogen, Grand Island, NY, USA). After propagation of the plasmids, they were purified using the Qiagen Plasmid Giga kit (Germantown, MD, USA) following the manufacturer’s instructions.

### Expression of the VVWR A27L vector

Gene expression was assessed by GenScript (Piscataway, NJ, USA). Gene coding for the A27 VVWR protein was subcloned into a His-Tag vector. Each recombinant plasmid was transiently transfected into 40 mL of suspension 293-6E cell culture. Cell culture supernatants (~30ml) were incubated with 0.2 ml Ni SephoroseTM 6 Fast Flow 0.2 ml (GE, Lot 10173458) for 3~4 hours to capture the target protein. After washing and elution with appropriate buffer, the eluted fraction was buffer exchanged to PBS, pH 7.2. The purified protein was analyzed by SDS‐PAGE and Western blot. A mouse-anti-His mAb (GeneScript, Piscataway, NJ, USA) was used as primary antibody.

### Mice

Female 4–6 week-old BALB/c mice were purchased from Charles River (Wilmington, MA, USA). Care of the animals was in accordance with the guidelines from The National Institutes of Health (Bethesda, MD, USA), and the University of Puerto Rico Institutional Care and Use Committee (IACUC). Each experiment was performed at least three times, and each group contained four mice.

### Study design

The antigenic plasmid was formulated at a 1.0 mg/mL concentration of DNA in a 0.15M sodium citrate buffer and 0.25% bupivacaine. Experimental groups are shown in Table 1. Animals were immunized three times, two weeks apart with a dose of 100 mg of DNA plasmid coding for the VVWR A27 protein, with and without 50 nmoles of imiquimod or resiquimod (Invivogen, San Diego, CA). Also, Naïve and backbone mice groups were included as controls. Blood was drawn from each animal one-week after the third immunization, in which animals were sacrificed by cervical dislocation, and spleens removed for analyses.

**Table 1:** Study design.

Groups	DNA Construct (μg)	Imiquimod (nmoles)	Resiquimod (nmoles)
Naïve	0	0	0
pVax1	100	0	0
pA27L	100	0	0
pA27L + imiquimod	100	50	0
pA27L + resiquimod	100	0	50
pA27LOPT	100	0	0
pA27LOPT + imiquimod	100	50	0

Experimental groups used on this study: 1. Naïve (negative control), 2. pVax1 (backbone control), 3. pA27L (100 μg DNA antigen), 4. pA27L+I (100 μg DNA antigen + 50 nmoles of imiquimod), 5. pA27L+R (100 μg DNA antigen + 50 nmoles of resiquimod), 6. pA27LOPT (100 μg DNA antigen) 7. pA27LOPT+I (100 μg DNA antigen + 50nmoles of imiquimod).

### Synthetic peptides

The peptides used in this study were derived from the sequence of the VVWR A27 protein, and synthesized as 3-mer overlapped, 15-mer amino acids by JPT Peptide Technologies (Berlin, Germany). These were prepared as both: 1) a peptide pool, and 2) as individual peptides for epitope mapping. All peptides were diluted to a concentration of 0.5 mg/mL in culture medium, and stored at -20°C.

### ELISPOT

Capture anti-mouse IFN-γ antibody (R&D Systems, Minneapolis, MN, USA) was coated by overnight incubation at 4°C onto High-Protein Binding IP 96-well Multiscreen TM plates (Millipore, Bedford, MA, USA). The plates were washed and blocked with 1% BSA. Then, 2 x 10^5^ spleen cells were added to each well in complete medium, and stimulated overnight at 37°C, in 5% CO_2_ with the VVWR A27 peptides (JPT Peptide Technologies, Berlin, Germany). Concanavalin A (Con A, 5 mg/mL; Sigma-Aldrich, St. Louis MO, USA), and media were used as positive and negative controls, respectively. After 24 hours of stimulation, the plates were washed and incubated overnight at 4°C in the presence of biotinilated anti-mouse IFN-γ antibody. The next day, plates were washed and streptavidin-alkaline phosphatase added to each well for two hours at room temperature. The plates were washed again, and 5-Bromo-4-Chloro-3’ Indolylphosphate p-Toluidine Salt (BCIP) and Nitro Blue Tetrazololium Chloride (NBT) (R and D Systems, Minneapolis, MN) was added to each well for 30 min at room temperature. Subsequently, plates were rinsed with distilled water, and dried inverted at room temperature. Spots were quantified by an automated ELISPOT reader system (CTL analyzers, Cleveland OH, USA) with the ImmunoSpot software. The mean number of spots from triplicate wells was adjusted to 1x10^6^ splenocytes. Antigen-specific responses to IFN-γ were obtained after subtracting the number of spots formed in the wells containing the control medium from the spots formed in response to the peptides. ELISPOT data are expressed as mean ± standard error of the mean.

### Epitope mapping

Individual 3-mer overlapped, 15-mer peptides from VVWR antigens were used as specific stimulators, in an ELISPOT assay designed to map the MHC I dominant and sub-dominant regions. The epitope that produced a positive signal was identified by the appearance of spots in its ELISPOT well.

### Humoral response by antibody determination

The humoral immune response was determined one week after the third vaccination by Enzyme-Linked Immunosorbent Assay (ELISA). Besides total IgG, we also measured the IgG_1_ (TH2-type), and IgG_2a_ (TH1-type) isotype responses. Serum samples were collected from blood after orbital bleeding of mice, as specified in the immunization schedule. Specifically, 1 μg/mL of the antigenic protein dissolved in carbonate buffer 0.05M was incubated overnight on Maxisorp (Millipore, Bedford, MA) plates at 4°C. Then, after washing the plate with PBS, the non-specific reactivity on the wells was blocked by incubating the plates in BSA-supplemented PBS (PBSB, PBS +1% BSA) for 1 hour at 37°C. Serum was incubated as duplicate serial dilutions in PBSB for two hours at room temperature. After washing, HRP-conjugated goat anti-mouse IgG, or IgG_1_, or IgG_2a_ (Jackson Immunoresearch, West Grove, PA, USA) was added to the wells at a 1:2500 dilution, and incubated for 1 hour at room temperature. After washing, absorption produced by the attached antibody was measured by the addition of the substrate 3,3′,5,5′-Tetramethylbenzidine (TMB). Reaction was stopped by adding 100 mL of 2.5 M sulfuric acid per well, and absorption was determined at 450 nm in an ELISA reader.

### Cytokine determination

Cytokine profile was determined by ELISA, using the commercially available Quantikine Mouse IFN-γ and IL-4 immunoassays, following the manufacturer’s protocol (R&D Systems, Minneapolis, MN, USA). Briefly, mouse splenocytes were incubated in a 96-well plate at 2 x 10^5^ cells/well in complete medium, and stimulated overnight at 37°C, in 5% CO_2_ with the VVWR A27L peptides (JPT Peptide Technologies, Berlin, Germany). Concanavalin A (Con A, 5 mg/mL; Sigma-Aldrich, St. Louis MO, USA) and media were used as positive and negative controls, respectively. Then, 50 μL of each sample supernatant were transferred to another plate and incubated with 50 μL of Assay Diluent for 2 hours at room temperature. After washing, 100 μL of Mouse IFN-γ or IL-4 conjugate were added to each well, incubated for 2 hours at room temperature, and washed again. Then, 100 μL of Substrate Solution were added to each well and incubated for 30 min at room temperature. After stopping the reaction, the optical density of each well was determined by a microplate reader at 450 nm. The concentration of the each sample was obtained after correlating with a standard curve.

### Statistical analyses

Immunization studies show data from experiments that were repeated at least three times. The immune responses among groups of mice are presented as the mean ± standard error of the mean (SEM). The statistical significance of differences among groups was determined by one-way ANOVA followed by the Tukey’s test when the null model was rejected, using the GraphPad Prism (La Jolla, CA, USA) and JMP 10.0 statistical software (SAS, Institute Inc., Cary, NC, USA. A *p* value of less than 0.05 was considered significant. Prior to testing the null model, data were evaluated for normality with the Shapiro-Wilk’s test and equality of variance with the Brown-Forsythe tests. Data transformation was performed (either log10(x) or sqrt(x+3/8) to normalize the data when necessary. If test of equality of variance posterior to evaluating alternative transformation was still significant, the best transformation was used and the Welsh ANOVA test was used to allow for the standard deviation not being equal. However, posterior to transformation data there was little evidence of deviance from normality and groups were mainly homoscedastic. A series of evaluation of the influence of sample size on results were performed, this measure of elasticity (random change in samples on parameter estimates) showed that the results were robust (simulation not shown here).

## Results

### Restriction digestion of pA27L

**Fig 1:**
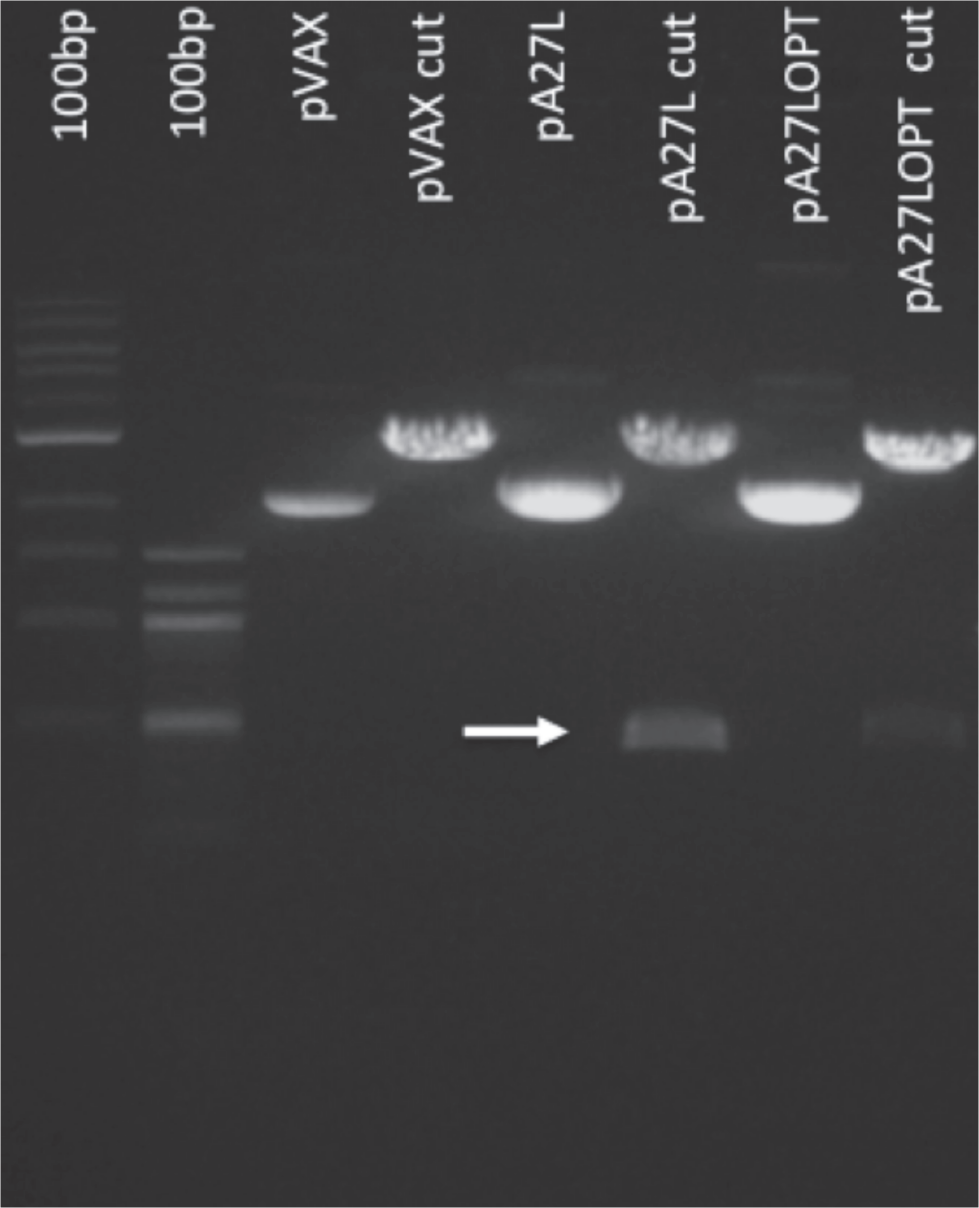
Enzymatic digestion of the pA27L plasmid. The A27L and A2L OPT gene was enzymatically digested with HindIII and NotI. Lane 1, 1kb DNA ladder; Lane 2, 100bp DNA ladder; Lane 3, pVax1 uncut; Lane 4 pVax1 cut; Lane 5, pA27L uncut; Lane 6, pA27L cut; Lane 7, pA27LOPT uncut; Lane 8, pA27LOPT cut. The expected band of 434bp corresponding to the A27L gene is highlighted with an arrow.

The pA27L and pA27LOPT plasmids extracted from *E*. *coli* TOP10 transformed bacteria (Invitrogen, Grand Island, NY, USA) were enzymatically digested with HindIII and NotI (New England Biolabs, Ipswich, MA, USA). A band of 434 base pairs corresponding to the expected A27L gene was obtained, based on the estimated size by the Bioinformatics Software MacVector (Cary, NC, USA) (Fig 1).

### Protein expression

**Fig 2:**
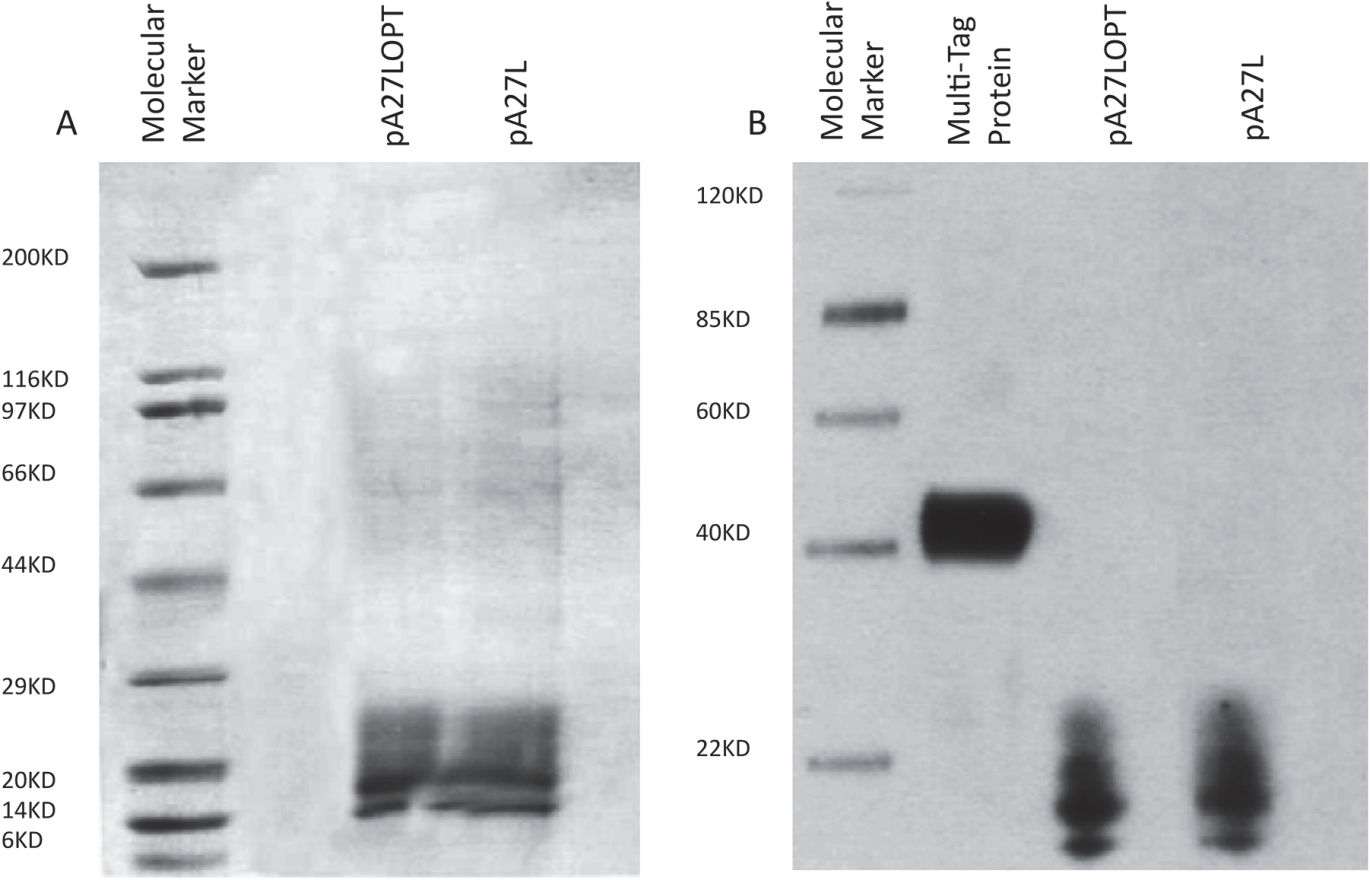
Antigen expression studies determined by Western Blot analysis. DNA constructs were tested for expression by (A) SDS-PAGE electrophoresis and (B) Western blot analyses. Proteins were purified under reducing conditions and detected using an anti-His mAb.

The A27 protein was successfully expressed and purified from pA27L and pA27LOPT plasmids (S1 File) in suspension 293-6E cell culture. The purified protein was detected by SDS-PAGE and Western blot analyses (Fig 2). The estimated molecular weights of the A27 protein is ~16kDa according to the Bioinformatics Software MacVector (Cary, NC, USA).

### VVWR A27-specific IFN-γ stimulation

We assessed the immunomodulatory effect of imiquimod and resiquimod after vaccinating BALB/c mice three times, two weeks apart, using the pA27L antigenic plasmid formulated as stated in Table 1.

**Fig 3:**
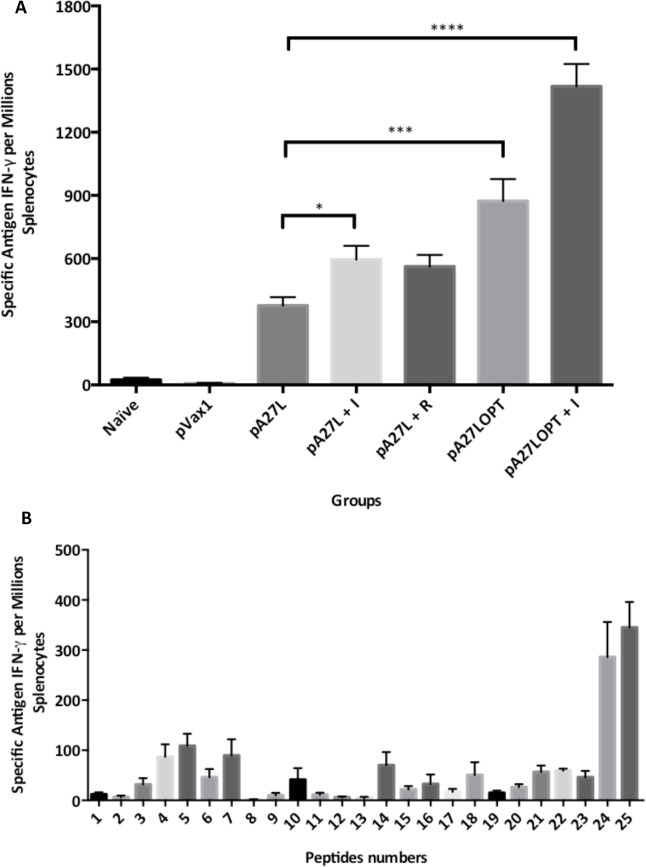
A27L-specific cell-mediated immune response against recombinant VVWR determined by ELISPOT analysis. (A) Each group consisted of four female Balb/C mice immunized three times, two weeks apart via intramuscular injection as follows: Naïve (negative control), pVax1 (empty vector backbone control), pA27L (VVWR envelope protein), pA27L+I (VVWR envelope protein + imiquimod), pA27L + R (VVWR envelope protein + resiquimod), pA27LOPT (optimized sequence of the A27L gene) and pA27LOPT+I (the optimized sequence of A27L + imiquimod). (B) Identification of A27L dominant epitopes. Antigen-specific IFN-γ ELISPOT in splenocytes from mice (immunized three times, two weeks apart via intramuscular injection) in response to VVWR A27 individual 3-mer overlapped 15-mer overlapping peptides. Each experiment was performed three times and the immune responses among groups of mice are presented as the mean ± standard error of the mean (SEM). A *p* value of less than 0.05 was considered significant.

The cellular response was studied from splenocytes pools obtained from each individual group of mice described in Table 1, after determining the frequencies of A27-specific IFN-γ-producing cells by ELISPOT analysis (Fig 3A). Specifically, one week after the last immunization, splenocytes pools of each group of mice were stimulated, using a pool of 3-mer overlapped 15-mer peptides representing the entire sequence of the VVWR A27protein.

Our data indicate that the mean frequency of the spot-forming cells (SFC) per million splenocytes was 2.6 ± 0.6, 2.0 ± 0.7, 377.1 ± 39.7, 595.0 ± 65.6, 562.5 ± 55.1, 873.6 ± 104.5 and 1418 ± 105.8 corresponding to Naïve, pVax1, pA27L, pA27L+I, pA27L+R, pA27LOPT and pA27LOPT+I respectively. A significant difference in the IFN-γ production was noted among groups (ANOVA test F_6, 156_ = 170.3, p < 0.0001, analysis performed on the X = Ln(X+1). The Naïve group serves to provide the baseline levels of IFN-γ, while the pVax1 control shows any possible contribution from the vector backbone (Fig 3A). A strong adjuvant effect in DNA immunization with imiquimod was demonstrated after observing a significant 1.6-fold increase in the amount of IFN-γ spot forming cells per million splenocytes in pA27L+I and a 3.8-fold in pA27LOPT+I immunized group, compared to animals immunized with pA27L. These data demonstrate the immunomodulatory ability of the adjuvant to significantly enhance an immune response, increasing the secretion of IFN-γ by T cells specific for the pA27L antigen.

### Epitope mapping

Then, we decided to track the dominant epitopes under these immunization conditions (Table 1). For this purpose, splenocytes isolated from mice immunized with pA27L were stimulated as described before, to measure the frequencies of A27-specific IFN-γ-producing cells by ELISPOT analysis. After testing 25 individual peptides, our data (Fig 3B) mapped two dominant regions: 1) A27L #24, 2) A27L #25. The mean frequencies of spot forming cells (SFC) per million splenocytes for each of these peptides were 286.1 ± 70 and 345 ± 51, respectively. A significant difference in the IFN-γ production was noted among different peptides (ANOVA test F_24, 137_ = 6.431, p < 0.0001, analysis performed on the X = log(X*10). Specifically we found significant differences in the frequency of IFN-γ-producing cells individually stimulated with A27L #24 and A27L #25 compare with the rest of the peptides (S1 Table).

### Cytokine profiling

**Fig 4:**
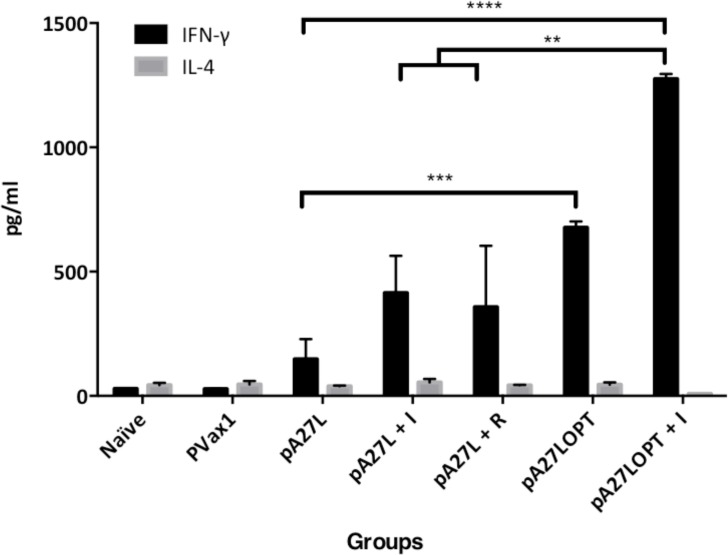
A27L-specific cytokine profile against recombinant VVWR A27L by ELISA. Each group consisted of four female BALB/c mice immunized three times, two weeks apart via intramuscular injection with pA27L, pA27L+I, pA27L+R, pA27LOPT, pA27LOPT+I. Naïve and pVax1 were used as controls. A week after the last immunization, splenocytes from each group of mice were stimulated overnight with VVWR A27-overlapping peptides, and the cytokine-containing supernatants were examined by ELISA. The plate was analyzed by scanning the absorption at 450 nm. The immune responses among groups of mice are presented as the mean ± standard error of the mean (SEM) of at least three independent experiments. A *p* value of less than 0.05 was considered significant.

Cytokines are known to have a role polarizing immunity towards TH1 or TH2. Specifically, IL-4 is recognized as the main cytokine responsible for generation of a TH2 response, whereas IFN-γ polarizes immunity towards a TH1 response. For this reason, we decided to investigate the cytokine profile from the supernatants of antigen-stimulated splenocytes from mice immunized with our vaccination cocktails. A significant difference in the cytokine profile was noted among groups (Welch’s ANOVA test F_6, 7.28_ = 1488.1, p < 0.0001, analysis performed on the sqrt(x +3/8). Our cytokine profile analysis shows that lymphocytes from mice immunized with pA27L produced 148.2 ± 86.8 pg/mL of IFN-γ, while lymphocytes from mice immunized with pA27L+I produced 414 ± 100.3 pg/mL. Moreover, the pA27LOPT immunized mice produced 678 ± 24.81 pg/mL compared to the pA27LOPT+I that produced 1275 ± 20.27 pg/mL. These data demonstrate a significant increase in IFN-γ on mice immunized with pA27LOPT+I, versus those immunized with antigen alone (pA27L), antigen plus imquimod (pA27L+I) and antigen plus resiquimod (pA27L + R) (Fig 4). This increased cytokine secretion was not observed when lymphocytes were tested for IL-4, which was detectable in only minimal amounts. Moreover, no significant difference was noted among groups in the amount of IL-4 (ANOVA F_6, 20_ = 2.001, p = 0.11). These data show a role of our vaccine formulation inducing a TH1-biased immune response.

### Humoral responses

**Fig 5:**
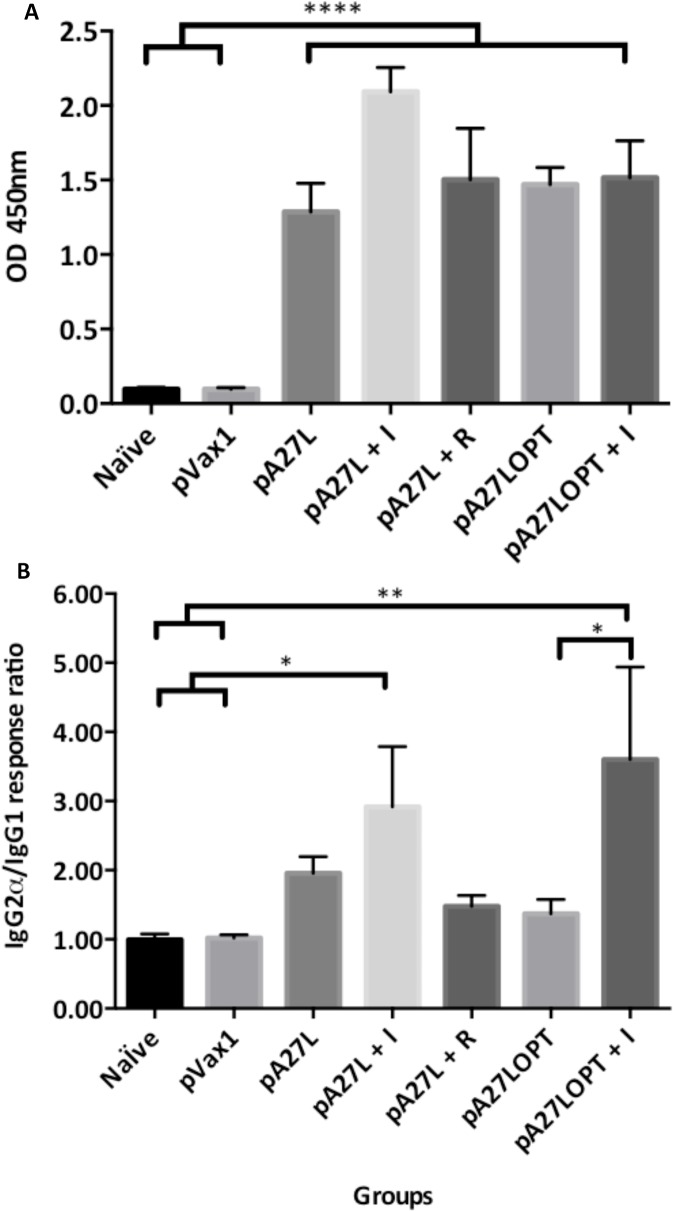
Humoral-mediated immune response against recombinant VVWR A27L by ELISA. A. Total IgG response after DNA immunization of four mice three times, two weeks apart with A27L, A27L+I, pA27L+R, pA27LOPT, pA27LOPT+I. Naïve and pVax1 were used as controls, One week after the last immunization, sera from each group of mice were diluted at 1:400, and incubated in a 96-well plate previously coated with recombinant VVWR A27 protein. B. Antigen-specific production of each IgG subtype shown was detected from each group. The plate was analyzed by scanning the absorption at 450 nm and the immune responses among groups of mice are presented as the mean ± standard error of the mean (SEM) of at least three independent experiment. A *p* value of less than 0.05 was considered significant.

We examined the impact caused by imiquimod improving the A27-specific humoral response (Fig 5A). For this purpose, we designed an indirect ELISA using as an antigen the VVWR A27 protein, and an antibody against mouse total IgG. A modest enhancement in the humoral response was observed after vaccination. Specifically, an increase in the total IgG production was obtained in animals immunized with pA27L, pA27L+I, pA27L+R, pA27LOPT and pA27LOPT+I. These groups did not show a significant increase among them. However, they showed a significant increase compared to the Naïve and pVax1 groups (means comparison using the Tukey-Kramer HSD test, significant value when p<0.05). The significant difference among groups was tested using the ANOVA test F_6, 42_ = 57.50, p < 0.0001, analysis performed on the X = log(X*10).

### Antibody isotyping

The relative proportion of the subtypes of antibodies produced either after an infection or an immunization plays a role in the outcome of a humoral response. On one hand, antigens that preferably stimulate the production of opsonizing IgG2a antibodies support a TH1 type response, whereas preferred production of neutralizing IgG1 antibodies supports a TH2 type response. For this reason we designed an indirect ELISA protocol, to study how the ratios of IgG2a versus IgG1 were affected by our vaccine formulation.

We found a ratio greater than one (1.00) for all immunized groups. Moreover, after the third immunization, the antibody IgG2a/IgG1 ratio increased 1.5-fold in animals immunized with pA27L+I compared to animals immunized with pA27L. A significant difference in the ratios of IgG2a versus IgG1 was noted among groups (ANOVA test F_6, 28_ = 4.765, p = 0.0018). Furthermore, the pA27LOPT+I group demonstrates a significant difference compared to both, the control groups (Naïve and pVax1) and the pA27LOPT (Fig 5B).

## Discussion

The fact that smallpox vaccination campaigns have been eliminated has left the United States incompletely protected against smallpox. The current preventive measures to protect the population in case of smallpox outbreak is the licensed vaccine, which consists of live vaccinia virus. With the intent to develop a virus-free DNA vaccine, safe for the entire population, we decided to test the effect of an immunomodulator, enhancing the immune response of a DNA vaccine coding for the VVWR A27L gene.

Ideally, a vaccine should be designed to be as simple as possible, minimizing its components, storage, transportation and manipulation. In this regard, although many researchers have shown smallpox DNA vaccines to be effective only when multiple vaccinia virus antigens are combined [43], another group has shown viral protection with a vaccine consisting of only one gene coding for A27 delivered by a viral vector [44]. This finding is significant since it reduces the component that is activating the immune response to only one antigen. We took this information into consideration when choosing A27L as part of our vaccine formulation.

The vaccinia virus antigen used in this study was selected based on its immunogenicity, viral function, viral localization, and stage of the viral cycle in which is produced. Furthermore, A27L has been used in several studies where viral protection was shown. On this subject, in a nonhuman primate model, A27L combined with A33R, L1R and B5R was shown to protect against a monkeypox challenge [43]. Moreover, as stated before, an A27L-recombinant adenovirus vaccine administered as a single intramuscular injection was shown to protect mice against a lethal intranasal poxviral challenge [44]. Therefore, as a first step, it is worth studying if the A27-specific immune response, in a virus-free DNA vaccine formulation, could be significantly enhanced by an immune-modulator like imiquimod or resiquimod.

It is well accepted that the cell-mediated immune response has a crucial role in the delay of disease progression, while the humoral response takes place and clears the virus [45, 46]. In this regard, our data shows that the increase in the frequency of cells producing gamma interferon was significantly different for animals immunized with pA27L, and animals immunized with pA27L+I or pA27LOPT+I. This might shed new ideas on the identification of the mechanisms involved in the immune response and provides novel resources for immune protection. Imiquimod is a TLR-7 agonist, its response is MyD88-dependent, directing towards the activation of NF-κB [32]. This fact provides important information correlating, at the molecular level, the activation pathways of the TLR-7 with the efficacy of the immune responses induced by our vaccine formulation.

All antigen-immunized animals were able to generate humoral responses. This is not surprising since A27L is known to induce antibodies [47]. However, the humoral response in animals immunized with pA27L+I showed a remarkable enhancement in the production of total IgG. In our experiments, the major difference was observed on serum antibody diluted 1:400. This data shows the capacity imiquimod enhancing the A27-specific humoral immune response. Moreover, although antibody isotype analysis shows an IgG2a/IgG1 ratio higher than 1 within all groups where pA27L was present, this ratio is significant in the group immunized with imiquimod (pA27LOPT+I) compared with non-adjuvant group (pA27LOPT). This demonstrates the adjuvant capacity of imiquimod enhancing a TH1-biased humoral response, which is known to be effective against viral infections.

In this article we have shown imiquimod to enhance the vaccinia virus-specific immune response by DNA-based immunization. The codon optimization of the A27L antigen improved the immunogenicity of the DNA vaccine. This information confirms research done by others investigators, which demonstrated that codon optimization of DNA sequence from mycobacterial [48], HIV [49], *Plasmodium falciparum* [50] among other antigens could improve protein expression and thereby enhance the immunogenicity of gene-based vaccines.

Previous studies from other groups have shown viral protection with a vaccine consisting of only one gene coding for A27. In their work, an A27L-recombinant adenoviral vector delivers the vaccine. As opposed to DNA immunization, this process occurs during an actual live viral infection, in which additional cellular and molecular events are activated. It is expected that, in the context of a real infection, other immune-activation pathways become engaged, producing a more complete and effective immune response. In fact, they successfully prove that A27L is sufficient protecting mice against a lethal intranasal poxviral challenge [44]. However, a gene delivered in a viral vector is detracting from our interest of generating a virus-free vaccine. For these reasons, we decided to test the immunogenic capacity of A27L in a virus-free DNA vaccine formulation.

Others group of investigators report the capacity of imiquimod and resiquimod on immune responses using DNA as the vaccine agent administered by particle-mediated immunotherapeutic delivery (PMID). They report an increased in both CD4^+^ and CD8^+^ T cell responses when these adjuvants are used in combination with PMID and genegun technology [51]. Our vaccine formulation showed an increase in both humoral and cellular response without the aid of additional equipment, delivery systems or live-vectors. Consequently, without affirming that our formulation protects against a vaccinia challenge, we expect it will work well in our viral-challenge studies, using mice as a model.

As indicated previously, imiquimod is an immunomodulator, responsible for inducing the production of type-I interferons [31]. The production of type-I interferons limits the cellular events of protein production in order to suppress the regulatory and proliferative capacity of the virus [52] at the expense of limiting the same cellular machineries. As stated before, DNA based vaccines have the advantage of emulating a viral infection, since antigens are produced within the same cell. Moreover, post-translational modifications are similar to those of a viral infection.

However, for these reasons, we believe that we might not be taking full advantage of the adjuvant capacity of Toll-like receptor agonists, and further optimization of the vaccination process is necessary, if these components are expected to be part of a vaccine formulation. Specifically, the adjuvant-mediated enhancement of the desired immune response could be compromised when DNA-based vaccines are combined with an adjuvant that induces an antiviral effect via activation of type-I interferon pathways. Therefore, although our vaccines formulations have shown to be effective in the stimulation of an immune response during DNA-vaccination, we understand that further optimization of the vaccination protocol could produce an even higher enhancement of the immune response. This should be explored in a future study.

The imiquimod amount used in this study was selected based on our previous experience with resiquimod formulated with another antigen. However, although we clearly show imiquimod to have a significant role as an adjuvant, a dose-response curve for imiquimod should identify its optimal concentration. Again, this should be explored in a future study. We expect this information to contribute and open new possibilities on the rational development of a vaccine used to defend against bioterror agents and emerging diseases.

## Supporting Information

S1 File Sequence of the A27L and A27LOPT gene. (DOCX)

S1 Table Sequence of the VVWR A27 peptides. These were prepared as a peptide pool, and also as individual peptides and are synthesized as 3-mer overlapped 15-mer overlapping amino acids by JPT Peptide Technologies (Berlin, Germany). All peptides were diluted to a concentration of 0.5mg/mL in culture medium, and stored at -20°C.
(DOCX)
