# Supplementary material for: Immunomodulator-Based Enhancement of Anti Smallpox Immune Responses
Source: PLoS One. 2015 Apr 13;10(4):e0123113. doi: 10.1371/journal.pone.0123113 (PMC4395221; doi:10.1371/journal.pone.0123113)
Supplement: S1 File — (DOCX) [file pone.0123113.s001.docx]

pA27L

GGATCCGCCGCCACCATGGATTGGACTTGGATCTTATTTTTAGTTGCTGCTGCTACTAGAGTTCATTCTCACCACCACCACCACCATGAGAATCTGTATTTTCAGGGAGACGGAACTCTTTTCCCCGGAGATGACGATCTTGCAATTCCAGCAACTGAATTTTTTTCTACAAAGGCTGCTAAAAAGCCAGAGGCTAAACGCGAAGCAATTGTTAAAGCCGATGAAGACGACAATGAGGAAACTCTCAAACAACGGCTAACTAATTTGGAAAAAAAGATTACTAATGTAACAACAAAGTTTGAACAAATAGAAAAGTGTTGTAAACGCAACGATGAAGTTCTATTTAGGTTGGAAAATCACGCTGAAACTCTAAGAGCGGCTATGATATCTCTGGCTAAAAAGATTGATGTTCAGACTGGACGGCGCCCATATGAGtgaTGAGCGGCCGC

pA27LOPT

GGATCCGCCGCCACCATGGACTGGACCTGGATTCTGTTCCTGGTGGCCGCTGCCACACGGGTGCACAGCCACCACCACCACCACCATGAGAATCTGTATTTTCAGGGAGACGGAACACTGTTCCCCGGCGACGACGACCTGGCCATCCCCGCCACCGAGTTCTTCAGCACCAAGGCCGCCAAGAAGCCCGAGGCCAAGCGCGAGGCCATCGTGAAGGCCGACGAGGACGACAACGAGGAAACCCTGAAGCAGAGACTGACCAACCTCGAGAAGAAAATCACCAACGTGACCACCAAGTTCGAGCAGATCGAGAAGTGCTGCAAGAGAAACGACGAGGTGCTGTTCAGACTGGAAAACCACGCCGAGACACTGAGAGCCGCCATGATCAGCCTGGCCAAGAAAATCGACGTGCAGACCGGCAGAAGGCCCTACGAGTGATGAGCGGCCGC
