# Supplementary material for: Immunomodulator-Based Enhancement of Anti Smallpox Immune Responses
Source: PLoS One. 2015 Apr 13;10(4):e0123113. doi: 10.1371/journal.pone.0123113 (PMC4395221; doi:10.1371/journal.pone.0123113)
Supplement: S1 Table — These were prepared as a peptide pool, and also as individual peptides and are synthesized as 3-mer overlapped 15-mer overlapping amino acids by JPT Peptide Technologies (Berlin, Germany). All peptides were diluted to a concentration of 0.5mg/mL in culture medium, and stored at -20°C. (DOCX) [file pone.0123113.s002.docx]

| Peptide number | Peptide Name | Peptide sequence |
| --- | --- | --- |
| 1 | VVWR A27L_001 | H-MDGTLFPGDDDLAIP-OH |
| 2 | VVWR A27L_002 | H-LFPGDDDLAIPATEF-OH |
| 3 | VVWR A27L_003 | H-DDDLAIPATEFFSTK-OH |
| 4 | VVWR A27L_004 | H-AIPATEFFSTKAAKK-OH |
| 5 | VVWR A27L_005 | H-TEFFSTKAAKKPEAK-OH |
| 6 | VVWR A27L_006 | H-STKAAKKPEAKREAI-OH |
| 7 | VVWR A27L_007 | H-AKKPEAKREAIVKAD-OH |
| 8 | VVWR A27L_008 | H-EAKREAIVKADEDDN-0H |
| 9 | VVWR A27L_009 | H-EAIVKADEDDNEETL-OH |
| 10 | VVWR A27L_010 | H-KADEDDNEETLKQRL-OH |
| 11 | VVWR A27L_011 | H-DDNEETLKQRLTNLE-OH |
| 12 | VVWR A27L_012 | H-ETLKQRLTNLEKKIT-OH |
| 13 | VVWR A27L_013 | H-QRLTNLEKKITNVTT-OH |
| 14 | VVWR A27L_014 | H-NLEKKITNVTTKFEQ-OH |
| 15 | VVWR A27L_015 | H-KITNVTTKFEQIEKC-OH |
| 16 | VVWR A27L_016 | H-VTTKFEQIEKCCKRN-OH |
| 17 | VVWR A27L_017 | H-FEQIEKCCKRNDEVL-OH |
| 18 | VVWR A27L_018 | H-EKCCKRNDEVLFRLE-OH |
| 19 | VVWR A27L_019 | H-KRNDEVLFRLENHAE-OH |
| 20 | VVWR A27L_020 | H-EVLFRLENHAETLRA-OH |
| 21 | VVWR A27L_021 | H-RLENHAETLRAAMIS-OH |
| 22 | VVWR A27L_022 | H-HAETLRAAMISLAKK-OH |
| 23 | VVWR A27L_023 | H-LRAAMISLAKKIDVQ-OH |
| 24 | VVWR A27L_024 | H-MISLAKKIDVQTGRR-OH |
| 25 | VVWR A27L_025 | H-LAKKIDVQTGRRPYE-OH |
